# Psychotherapy – A Sound Investments In (Mental) Health Capital?

**DOI:** 10.1093/eurpub/ckac129.683

**Published:** 2022-10-25

**Authors:** J Turunen, K Gluschkoff, J Kausto, S Selinheimo, A Koskinen, A Väänänen

**Affiliations:** Finnish Institute of Occupational Health, Helsinki, Finland

## Abstract

**Introduction:**

The concept of health capital views health as a form of capital that produces healthy time to individual. This stock of capital - health that is - can decrease or increase. The potential of psychotherapy as individual's investment to (mental) health capital has been rarely studied in population level.

**Objectives:**

The aim of our study is to shed light on the returns on individual-level investments in health capital. We consider the use of psychotherapy as an investment in health capital. This investment offers potential returns for individual as a higher level of subsequent income. However, these returns are potentially heterogenous: we aim to show to whom the use of psychotherapy is a sound investment in health capital.

**Methods:**

We model the effects of mental health, and subsequent treatments such as the use of psychotropics and psychotherapy on income using two-way fixed effects regression.

**Results:**

Preliminary results show that different parts of working-age population seem to have different potential returns related to the use of psychotherapy. These heterogenous effects are related to previously reported socioeconomic status related disparities: the level of human capital i.e. income and education play a role in the profitability of the individual level investment made in the health capital by the use of psychotherapy.

**Conclusions:**

The use of psychotherapy has heterogenous effects on the income of individuals. The potential of this investment to produce health capital varies with education, the level of income prior to the use of psychotherapy.

